# A MEMS IMU De-Noising Method Using Long Short Term Memory Recurrent Neural Networks (LSTM-RNN)

**DOI:** 10.3390/s18103470

**Published:** 2018-10-15

**Authors:** Changhui Jiang, Shuai Chen, Yuwei Chen, Boya Zhang, Ziyi Feng, Hui Zhou, Yuming Bo

**Affiliations:** 1 School of Automation, Nanjing University of Science and Technology, Nanjing 210094, China; changhui.jiang1992@gmail.com (C.J.); soochow_njust@sina.com (B.Z.); byming@mail.njust.edu.cn (Y.B.); 2 Centre of Excellence in Laser Scanning Research, Finnish Geospatial Research Institute (FGI), Geodeetinrinne 2, FI-02431 Kirkkonummi, Finland; yuwei.chen@nls.fi (Y.C.); ziyi.feng@nls.fi (Z.F.); 3 Department of Photogrammetry and Remote Sensing, Wuhan University, 129 Luoyu Road, Wuhan 430079, China; zhouhui@whu.edu.cn

**Keywords:** microelectromechanical systems, inertial measurement unit, long short term memory recurrent neural networks, artificial intelligence

## Abstract

Microelectromechanical Systems (MEMS) Inertial Measurement Unit (IMU) containing a three-orthogonal gyroscope and three-orthogonal accelerometer has been widely utilized in position and navigation, due to gradually improved accuracy and its small size and low cost. However, the errors of a MEMS IMU based standalone Inertial Navigation System (INS) will diverge over time dramatically, since there are various and nonlinear errors contained in the MEMS IMU measurements. Therefore, MEMS INS is usually integrated with a Global Positioning System (GPS) for providing reliable navigation solutions. The GPS receiver is able to generate stable and precise position and time information in open sky environment. However, under signal challenging conditions, for instance dense forests, city canyons, or mountain valleys, if the GPS signal is weak and even is blocked, the GPS receiver will fail to output reliable positioning information, and the integration system will fade to an INS standalone system. A number of effects have been devoted to improving the accuracy of INS, and de-nosing or modelling the random errors contained in the MEMS IMU have been demonstrated to be an effective way of improving MEMS INS performance. In this paper, an Artificial Intelligence (AI) method was proposed to de-noise the MEMS IMU output signals, specifically, a popular variant of Recurrent Neural Network (RNN) Long Short Term Memory (LSTM) RNN was employed to filter the MEMS gyroscope outputs, in which the signals were treated as time series. A MEMS IMU (MSI3200, manufactured by MT Microsystems Company, Shijiazhuang, China) was employed to test the proposed method, a 2 min raw gyroscope data with 400 Hz sampling rate was collected and employed in this testing. The results show that the standard deviation (STD) of the gyroscope data decreased by 60.3%, 37%, and 44.6% respectively compared with raw signals, and on the other way, the three-axis attitude errors decreased by 15.8%, 18.3% and 51.3% individually. Further, compared with an Auto Regressive and Moving Average (ARMA) model with fixed parameters, the STD of the three-axis gyroscope outputs decreased by 42.4%, 21.4% and 21.4%, and the attitude errors decreased by 47.6%, 42.3% and 52.0%. The results indicated that the de-noising scheme was effective for improving MEMS INS accuracy, and the proposed LSTM-RNN method was more preferable in this application.

## 1. Introduction

Global Navigation Satellite System (GNSS) and Inertial Navigation System (INS) have been set up in various vehicles and carriers for navigation and tracking [1,2,3,4,5]. A GNSS receiver is usually a chip, which is small, low-cost and precise. With continuously receiving the signal from the navigation satellites in orbit, the GNSS receiver is able to provide reliable and constant positioning, navigation and timing (PNT) information [6,7,8,9,10]. However, limited by the principle that at least four satellites are essential for computing positioning and velocity, the GNSS receiver will fail to work normally under challenging signal conditions [6,7,8,9,10]. The navigation signal transmits from the satellites a long way, and becomes too weak when reaching the ground, therefore, it can be easily blocked temporarily by the environment. For bridging the signal outages, Inertial Navigation System (INS) is employed to output positioning information during the signal outage. Traditional fiber or laser Inertial Measurement Units (IMU) are precise, but too big and expensive for vehicles or handheld devices [11,12,13].

Recently, Microelectromechanical Systems (MEMS) IMU has gained a boom in applications of position and navigation, especially, vehicles, handheld devices, and precise-guidance bombs, due to its low cost and small size brought by the advanced MEMS manufacturing technology [14,15,16]. With proper circuit and structure design, the accuracy of MEMS IMU has gradually improved [14,15,16]. Although, the MEMS IMU obtained a reduction in volume, and cost compared with conventional fiber or laser IMU, the MEMS IMU experiences more non-linear or random errors, which leads to the MEMS INS navigation solutions diverging dramatically over time [14,15,16,17,18]. Commonly, the GNSS receiver is employed as an outer aiding to calibrating the INS, thus, the GNSS and INS integrated navigation system is able provide reliable and continuous navigation solutions, even during short-term signal outage. During the outage, the problem is that the errors of INS increase quickly without outer sensors or reference aiding. Under this condition, the modeling or de-noising of the MEMS IMU outputs will be a key step to improve the accuracy of MEMS INS. Researchers are devoted to identifying and modeling the errors contained in the MEMS IMU raw signals, which can be divided into two parts: System errors and random errors. The system part refers to the bias, and scale factor errors. These errors can be calibrated or quantified by certain experiments in a laboratory. The calibration process has been investigated and reported by a number of researchers [19,20,21,22]. However, the random part can lead to the drifts and instabilities in bias or scale factor over time, which is the key component leading to the INS errors divergence [17,18,19,20,21,22,23,24,25,26].

Therefore, before deploying MEMS IMU for navigation, an accurate model of the random and systemic is requisite to ensure the accuracy. For identifying and modeling the random errors, researchers have devoted to proposing some de-nosing techniques in this random signals processing, and overall, the approaches can be divided to statistical modeling methods represented by Wavelet De-noising (WD), Allan Variance (AV), Auto Regressive and Moving Average (ARMA), and Artificial Intelligence (AI) methods represented by Support Vector Machine (SVM), Neural Networks (NN) [17,18,19,20,21,22,23,24,25,26]. Generally, the random errors contain high frequency (long-term) and low frequency (short-term) parts. Among statistical methods, the WD method performs significantly in removing the high frequency part [17,18,19,20,21,22,23,24,25,26], but it has restricted ability in removing low frequency errors. The AV method is another statistical method, and has been widely used in MEMS IMU errors analysis in time domain [17,18]. In the AV method, the stability of MEMS IIMU measurements are presented as a function of average time, and the intrinsic noise is described by five basic parts termed as: Quantization noise, angle random walk, bias instability, rate random walk, and rate ramp [17,18]. Usually, the AV method is employed to exploit the noise characteristics and obtain first-order Gauss Markov (GM) or ARMA parameters [17,18]. Traditional or conventional approaches are unsatisfactory for this application. Moreover, the unsatisfactory estimation or compensation of the random errors will lead to the failure to provide reliable navigation information estimation in short time. Another approach is AI methods including SVM and NN, which have been utilized in MEMS IMU modeling, and found to be better than other conventional methods [27,28,29,30,31,32,33,34,35,36]. These methods operate the signal de-noising or modeling as sequence prediction problem, and the MEMS IMU measurements are treated as time series.

Generally, in the data science community, sequence prediction problems have been around for a long period of time in a wide range of applications, including stock price prediction and sales pattern finding, language translation and speech recognization [37,38,39,40,41]. Recently, a new breakthrough has happened in the data science community, and a Long Short Term Memory Recurrent Neutral Networks (LSTM-RNN) has been proposed and has been demonstrated more effective for almost all of these sequence prediction problems [37,38,39,40,41]. Compared with conventional RNN, LSTM-RNN introduces the “gate structure” to address the long-term memory, which allows it to have the pattern of selectively remembering for a long time. This special design or structure makes it more suitable for predicting or processing time based series data. In this paper, the LSTM-RNN is incorporated in MEMS IMU gyroscope raw signal de-noising. LSTM-RNN has performed excellently in time series signal processing, for instance stock price prediction, speech single processing, and others [37,38,39,40,41]. A MEMS Inertial Measurement Unit (IMU) manufactured by MT Microsystems Company known as MSI2000 IMU is employed in the experiments for testing [42]. Firstly, a common ARMA is employed to process the raw signal, then the order and parameters are determined through the auto-correlation and partial correlation operation; secondly, a single LSTM and multi-layer LSTM are compared in the MEMS gyroscope raw signal de-nosing in aspects of average training loss, training time and de-noising performance; finally, the three-axis attitude errors of raw signals, ARMA, and LSTM-RNN are compared and analyzed.

The remainder of this paper is organized as: (1) Section 2 introduces the methods including Auto Regressive Moving Average Method (ARMA), and the proposed LSTM-RNN; (2) Section 3 presents the experiments, results and comparison (3) the following are the discussion, conclusion and future work.

## 2. Method

In this section, the conventional ARMA representing the statistical methods and the proposed LSTM-RNN representing AI methods are presented. The principles, basic equations and information flow are briefly introduced.

### 2.1. ARMA Model

As illustrated in previous papers [25,26], the following two steps are essential for setting up an ARMA model: (1) After the obtaining the raw gyroscope signals, auto-correlation and partial correlation are operated to characterize the noise and select the suitable time series model; (2) estimating the parameters of the ARMA model.

The auto-correlation of a signal is a product operation of the signal and a time-shifted version of the signal itself. Assuming r(t) is a random signal sequence, and the auto-correlation can be modelled as [25,26]:

(1) R(τ)=E(r(t)r(t+τ))

where, E(·) is the expectation operator, τ is the time delay or shift. The partial correlation process is defined as [25,26]:

(2) $$P\left(\tau \right)=\frac{\sum \left(r\left(t\right)-E\left(r\left(t\right)\right)\right)\left(r\left(t+\tau \right)-E\left(r\left(t+\tau \right)\right)\right)}{\sqrt{\sum {\left(r\left(t\right)-E\left(r\left(t\right)\right)\right)}^{2}\sum {\left(r\left(t+\tau \right)-E\left(r\left(t+\tau \right)\right)\right)}^{2}}}$$

where, E(·) is the expectation operator, τ is the time delay or shift.

The ARMA model is defined as:

(3) $$z\left(k\right)=\sum_{i=1}^{p}a_{i}z\left(k-i\right)+\sum_{j=1}^{q}b_{j}\varepsilon \left(k-j\right)+\varepsilon \left(k\right)$$

where, ε(k) is a zero mean and unknown white noise, p and q are the order of the ARMA model, z(k−i) is the input time series data, and the $a_{i}$ and $b_{j}$ are the related parameters, some approaches are published for obtaining the values of these parameters, for instance Kalman filter and the least square estimation method [25,26]. Generally, the auto-correlation and partial correlation function is used to decide the order of the AMRA model.

### 2.2. LSTM-RNN Method

**Figure 1:**
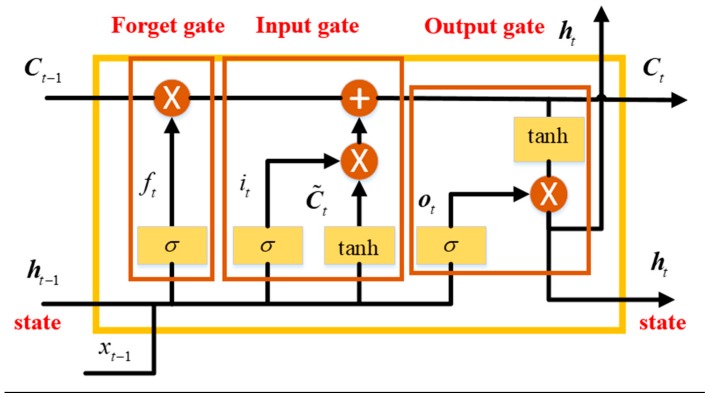
Basic structure of a Long Short Term Memory (LSTM) Unit.

Long Short Term Memory (LSTM) is a popular variant of the common Recurrent Neural Network (RNN). An RNN composed of LSTM units is often called an LSTM network. Different from RNN, new structure termed as “gate” is added to LSTM. Commonly, a LSTM unit is composed of a cell, an “input gate”, “output gate” and a “forget gate.” The basic structure of a single layer LSTM unit is shown as Figure 1. The cell remembers values over arbitrary time intervals and the three different gates regulate and control the flow of information into and out of the cell [38,39,40]. Following is the detailed description of the different gates and relative equations.

As illustrated in Figure 1, the first part of the LSTM is the “forget gate”, which is employed to decide what information is going to get thrown away from the cell state, the decision is made by a sigmoid layer called “forget gate layer”. $h_{t-1}$ and $x_{t}$ are input to the function, and outputs a value ranging from 0 to 1 for each number in the cell state $C_{t-1}$. The values represent the forgetting degree of each number in the cell state, and “1” represents “completely keep this” while “0” represents “completely get rid of this”. The operation equation $f_{t}$ is as:

(4) $$f_{t}=\sigma \left(W_{f}\cdot \left[h_{t-1},x_{t}\right]+b_{f}\right)$$

where, σ(·) is a sigmoid function, $W_{f}$ is the updating weights, $b_{f}$ is the bias, $h_{t-1}$ is the hidden state, and $x_{t}$ is the input vector.

After deciding the memory of the previous hidden state, and the second part is the “input gate”, which is utilized to decide what new information is going to be stored in the current cell state. This gate is composed of two parts: (1) A sigmoid layer to decide what values are going to be updated, the output values $i_{t}$ range from 0 to 1, and they represent the updating degree of each number in input; (2) another part is a *tanh* layer which creates a vector of new candidate values ${\tilde{C}}_{t}$, which will be added to the cell state after multiplied with the decision vector $i_{t}$. The relative equations are as following:

(5) $$i_{t}=\sigma \left(W_{i}\cdot \left[h_{t-1},x_{t}\right]+b_{i}\right)$$

(6) $${\tilde{C}}_{t}=\tanh\left(W_{C}\cdot \left[h_{t-1},x_{t}\right]+b_{C}\right)$$

where, σ(·) is a sigmoid function, $W_{i}$ is the updating weights, $b_{i}$ is the bias in the input gate, $h_{t-1}$ is the hidden state at time t−1, $W_{C}$ is the updating weights, $b_{C}$ is the bias, and $x_{t}$ is the input vector.

The last part is the “output gate”, which is employed to decide what is going to output, similarly, a sigmoid layer outputs values $o_{t}$, which is employed to decide what parts of the cell state will be output, then the cell state is put through a *tanh* function. After this operation, the cell state values are pushed to be between −1 and 1. Finally, the results are multiplied by the output of the sigmoid gate, and the output parts are decided. The related equations are as:

(7) $$o_{t}=\sigma \left(W_{o}\cdot \left[h_{t-1},x_{t}\right]+b_{o}\right)$$

(8) $$h_{t}=o_{t}*\tanh\left(C_{t}\right)$$

where, $W_{o}$ is the updating weights, and $b_{o}$ is the bias in the output gate, $C_{t}$ is the cell state at time t.

**Figure 2:**
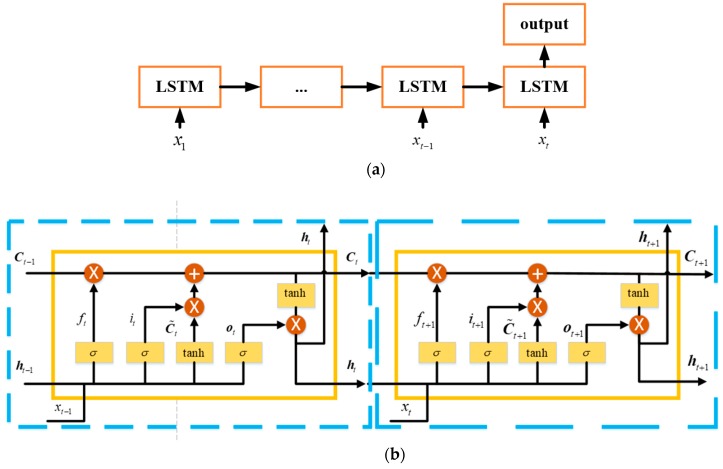
Working flow of the LSTM-RNN. (**a**) brief working flow of LSTM-RNN (Recurrent Neural Network); (**b**) a sequence of LSTM Unit.

The above Equations (4)–(8) describes the basic LSTM unit for RNN, which is just a single LSTM unit. Figure 2 presents a sequence of LSTM-RNN units in time domain. In Figure 2a, is a simple description of the LSTM-RNN working flow. The output of LSTM-RNN is decided by not only current state, but a long-term memory. Figure 2b gives the details. The cell state and hidden layer is covered to the next LSTM Unit, and the inner gate will decide the memory degree of past information. In addition, before it is employed for prediction, a training procure is necessary for determining the unknown parameters in the above Equations [37,38,39,40].

## 3. Experiments and Results

**Figure 3:**
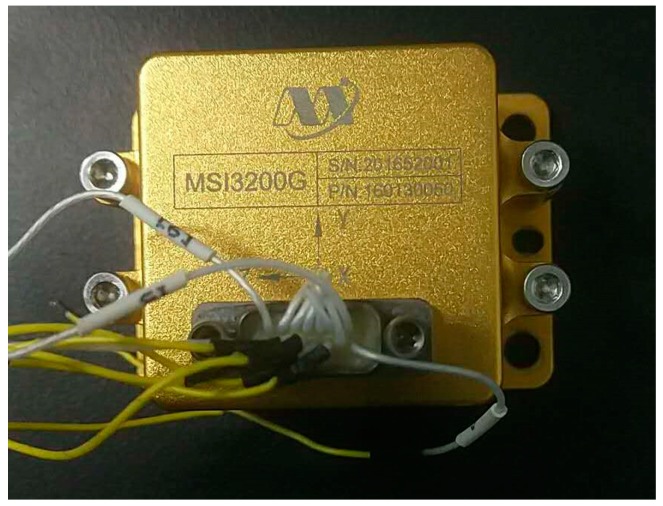
MSI3200 Inertial Measurement Unit.

**Figure 4:**
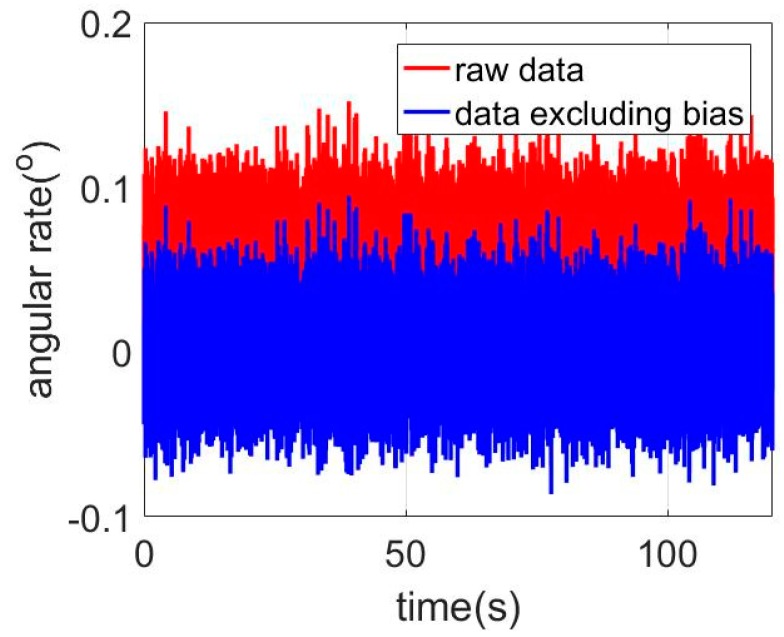
X-axis gyroscope signals.

This section will present experiments and the relative analyses for evaluating the performance of the proposed LSTM-RNN method. The laboratory experiments are conducted using the data which was collected from a MEMS IMU (MSI3200) manufactured by MT Microsystems Company, Shijiazhuang, China [29]. The real picture and the specifications of the IMU are as Figure 3 and Table 1 respectively. The gyroscope bias stability is ≤10°/h, and the random walk is $\leq 10{}^{\circ}/\sqrt{h}$. The accelerometer bias was 0.5 mg, and the bias stability was 0.5 mg. The IMU was placed statically on the table, and the sampling frequency was 400 Hz. Thus, the amount of data was 48,000. The gyroscope output data unit was in degree/s. The raw noisy data of the X-axis gyroscope output is shown as Figure 3 (red line representing the raw data), and the bias was reduced before modeling the errors. After removing the bias, the result is presented in Figure 4 (blue line representing the data excluding bias). Note that the program in this experiment was developed in Python with the Tensorflow package, which is operated in an Alienware R2 PC installed an i7 Intel CPU and 16 GB random memory.

The remainder of his section is divided into three parts: (1) The first part describes the results using the ARMA method to model the errors with presenting the auto-correction and partial correction results. The ARMA models are given according to the auto-correction and partial correction results. The standard deviation (STD) values of the signals are compared with the corresponding raw gyroscope signals; (2) the second part is the results using the LSTM-RNN to model the errors, and presenting the training time and prediction accuracy for different input vector length. Moreover, a multi-layer LSTM-RNN is designed and compared with a single-layer LSTM-RNN in terms training time, computation load and performance; (3) the last part presents the comparisons conducted between the ARMA and LSTM-RNN, including statistical results and position results.

### 3.1. Error Modeling Using ARMA

**Figure 5:**
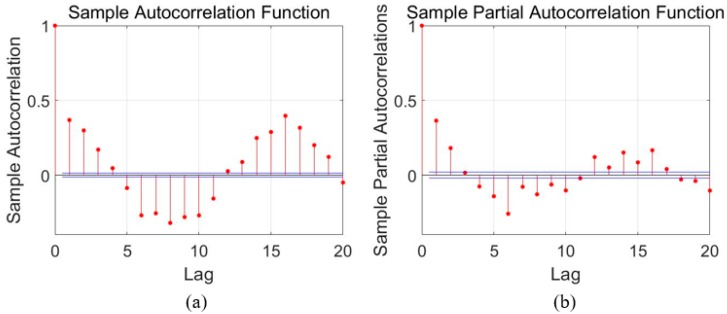
Auto-correlation and partial correlation analysis results of X-axis gyroscope signals. (**a**) autocorrelation analysis diagram; (**b**) Partial correlation analysis diagram.

**Figure 6:**
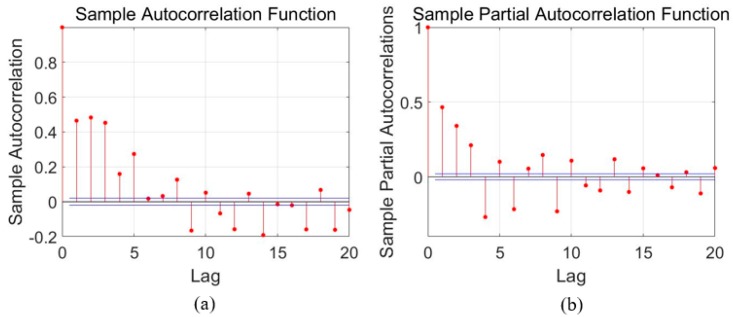
Auto-correlation and partial correlation analysis results of Y-axis gyroscope signals. (**a**) autocorrelation analysis diagram; (**b**) Partial correlation analysis diagram.

**Figure 7:**
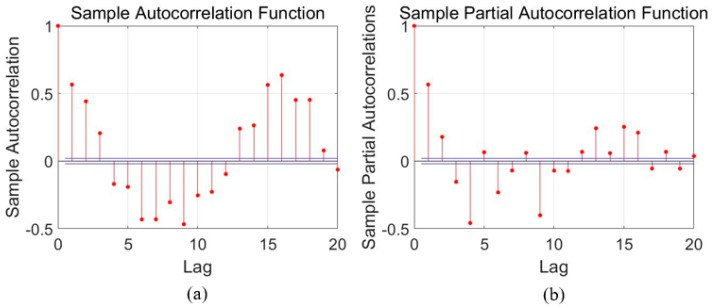
Auto-correlation and partial correlation analysis results of Z-axis gyroscope signals. (**a**) autocorrelation analysis diagram; (**b**) Partial correlation analysis diagram.

For time series analysis, Auto-Correlation Function (ACF) and Partial Auto-Correlation Function (PACF) characteristics are usually employed to select the proper model. As aforementioned in Section 2, the ACF and PACF are presented as Equations (1) and (2), and then Figure 4, Figure 5 and Figure 6 show the ACF and PACF results of the X-axis, Y-axis, and Z-axis gyroscope raw signals respectively, which are processed according to the Equations (1) and (2). From Figure 5, Figure 6 and Figure 7, it is evident and obvious that ACF and PACF of the three-axis gyroscope are tail off. Thus, the ARMA model is suitable for this application, and the order of the ARMA model is determined using the results. More details about parameters determination can be found in the references [25,26]. Therefore, ARMA models for these three-axis gyroscope signals are presented as:

(9) z(k)=0.3475z(k−1)+0.163z(k−2)−0.0508ε(k−1)+ε(k)

(10) z(k)=0.5065z(k−1)+0.2583z(k−2)−0.2371ε(k−1)+ε(k)

(11) z(k)=0.3309z(k−1)+0.2592z(k−2)+0.132ε(k−1)+ε(k)

where, the z(k) is the data at time ε(k) is the white noise at time k. The results from the ARMA is listed in Table 2. Compared with raw signal, the standard deviation (STD) of the three-axis gyroscope outputs decrease by 31.1%, 20.0% and 25.0%. The results show the ARMA performs effectively for de-noising MEMS gyroscope raw signals. Specifically, the parameters or orders are fixed in this experiment. 

### 3.2. Error Modeling Using LSTM-RNN

In this proposed LSTM-RNN method, the employed MEMS gyroscope dataset is labeled as $\left[x_{1},x_{2},\ldots ,x_{N}\right]$, the subscript N is termed as the amount of the IMU data samples. The dataset is divided into a training and testing part. The training part is used to build the model and the testing part is used to verify the model. The input data vector for training is defined as:

(12) $$Input_{i}=\left[x_{i},x_{i+1},\ldots ,x_{i+step}\right],i\in \left[1,N-step\right]$$

The output data vector is defined as:

(13) $$Onput_{i}=\left[x_{i+step+1}\right],i\in \left[1,N-step\right]$$

In above equations, the variable step is the length of the input data vector for training procedure. Suitable values of the input vector length step is identified to realize a tradeoff between training time and the prediction performance. Table 3 shows the three axis gyroscope data training results and standard deviation (STD) of the prediction. In this test, the 5, 10, 15, 20 and 30 are the selected values of the vector length. Table 4 shows the comparison results. The training dataset length is 1000, and the testing dataset length is 48,000 (2 min with 400 Hz sampling rate). The specifications of the LSTM-RNN are presented in Table 5. The consumption time increases with the input vector length, and the STD values decrease first, and then increase. Hence, 20 is selected as the length of input vector, which is the best tradeoff between the STD and the computation time.

As shown in Table 5, the noises are considerably decreased using the LSTM-RNN, and the STD values decrease by 60.3%, 37%, and 44.6%. As aforementioned, the training epoch was set to 50, and a multi-layer LSTM-RNN was designed and compared with single LSTM-RNN. Table 6 shows the results. The multi-layer LSTM-RNN (two hidden units) has a lower average training loss at epoch of 50, which decreases by 55.4%, 34.2% and 32.1%, However, it seems that there is no obvious advance in filtering performance. The STD values of the filters data have no reduction, and are even a little higher. The operation time consumption is almost twice that of single LSTM-RNN.

**Figure 8:**
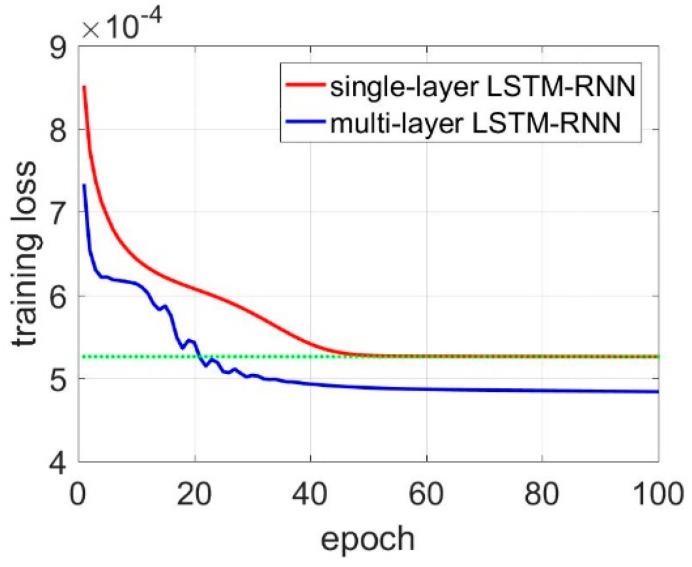
Training loss comparison between single-layer LSTM and multi-layer LSTM.

Figure 8 shows the training loss comparison of single-layer LSTM RNN and multi-layer LSTM-RNN, and they have identical accuracy at the 20th epochs. Thus, multi-layer LSTM-RNN was trained with 20 epochs, and Table 7 shows the results for the multi-layer LSTM-RNN. The results were compared with single LSTM-RNN with 50 training epochs. The average training losses have a slight increase, and the STD values are almost identical to that of multi-layer LSTM-RNN with 50 training epochs. However, compared with the single LSTM-RNN, the time consumption of multi-layer LSTM-RNN is less than that of single LSTM-RNN, which is initialed by the training epochs reducing in multi-layer LSTM-RNN. In aspects of the STD values, the de-noised three-axis gyroscope outputs have an improvement of 22.4%, 9.1%, and 22.6% respectively compared with single-layer LSTM-RNN trained after 50 epochs. This decline in accuracy means the multi-layer LSTM-RNN with fewer training epochs have weaker generation ability, since the multi-layer LSTM-RNN has more parameters which need more training epochs. Thus, while the training epochs are set to 20, the multi-layer has slightly worse STD values compared with the single-layer LSTM-RNN.

### 3.3. Comparisons of ARMA and LSTM-RNN

This part presents the comparisons between ARMA and LSTM-RNN. Table 8 shows the STD results from the ARMA and LSTM-RNN de-noising methods. Compared with raw signals, STD values of the three-axis gyroscope data from the ARMA method perform a 31.2%, 20.0% and 25.0% improvement, and the STD values of the single-layer LSTM-RNN de-noised signals decrease by 42.4%, 21.4% and 21.4% respectively. With the same MEMS IMU dataset, the LSTM-RNN has an obvious improvement of 42.3%, 21.4% and 26.2% respectively for the three-axis gyroscope dataset.

**Figure 9:**
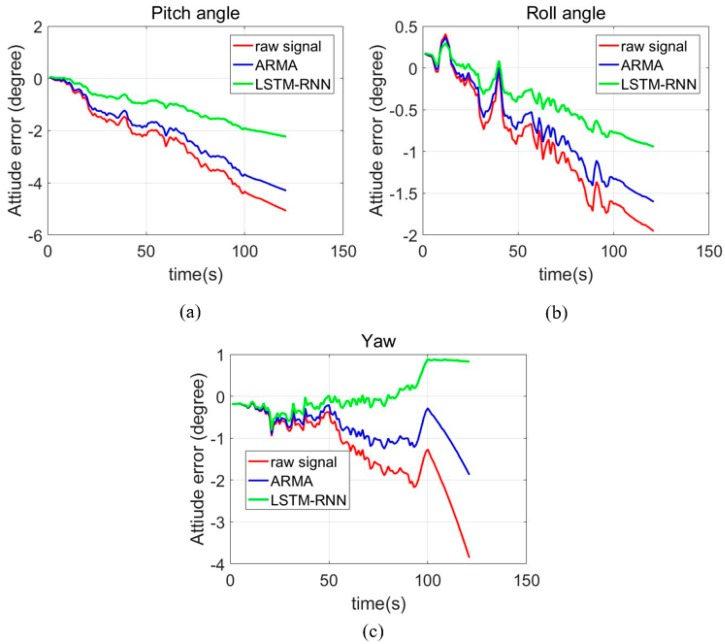
LSTM-RNN MEMS IMU attitude errors. (**a**) Pitch angle; (**b**) Roll angle; (**c**) Yaw angle

Further, Figure 9 shows the attitude errors of the ARMA and LSTM-RNN de-noised MEMS IMU data. In the Figure 9, the blue line represents the position errors of the raw signals from MEMS IMU, the green line represents the position errors of AMMA de-noised MEMS IMU, and the red line represents the position errors of the designed single LSTM-RNN de-noised MEMS IMU. Table 9 shows the maximum errors of the three-axis gyroscope, compared with raw signal. The pitch, roll angle and yaw angle errors decreased by 15.8%, 18.3% and 51.3% respectively. Specifically, the pitch error decreases from −5.07° to −4.27°, the roll error decreases from −1.95° to −1.60°, and the yaw angle error decreases from −3.85° to −1.87°. Moreover, the errors from signals de-noised by LSTM-RNN decreased by 47.6%, 42.3% and 52.0%, further compared with ARMA results. To be specific, the pitch, roll and yaw angles have an extra improvement of 2.04°, 0.66° and 0.96° compared with that of ARMA. The ARMA employed in this experiment was operated with fixed parameters with selected part of the dataset. Thus, in the testing dataset, more feasible parameters are available and suitable for better performance. However, the single-layer LSTM and ARMA were tested with the identical dataset, this might demonstrate that LSTM-RNN have better generation ability in this application. This is what we think might account for the accuracy improvement of the single-layer LSTM-RNN compared with the common ARMA method. In addition, the yaw errors from the LSTM-RNN de-noised signals have an upward trend which is different from the errors from the ARMA and raw signals. We think the principle of the LSTM-RNN may account for this, and the specifications need more and further investigation. Overall, the results demonstrate the effectiveness of LSTM-RNN in MEMS gyroscope signals de-nosing.

## 4. Discussion

1. In this paper, limited by the computing capacity of the employed computer, the LSTM-RNN had a limited amount of layers, which might have a negative influence on the generation ability of LSTN-RNN and the prediction performance in the long term.
2. In this paper, just one of the RNN variants LSTM-RNN were employed and evaluated in this application, and it has significant meaning to explore different LSTM-RNN structures more suitable for MEMS IMU errors modeling and de-noising.
3. This method was tested only using static data, and dynamic trajectory data should be included for fully evaluating the proposed method. The noise characteristics in dynamic environment may be different from that in dynamics.

## 5. Conclusions

This paper discussed a LSTM-RNN based MEMS IMU errors modelling method. A MEMS IMU (MSI 3200) was employed for testing the proposed method. Through the comparisons, three major conclusions were drawn as: (1) LSTM-RNN outperformed the ARMA in this application. Compared with the ARMA model, the standard deviation of the single LSTM-RNN de-noised signals decreased by 42.4%, 21.4% and 21.4% respectively, and the attitude errors decreased by 47.6%, 42.3% and 52.0%; (2) multi-layer LSTM-RNN was able to realize the settled average training loss with less training epochs. However, the multi-layer LSTM-RNN did not outperform the single LSTM-RNN in standard deviation values of the prediction. When the training epoch was set to 20, the multi-layer had a slightly better prediction accuracy with less computation time than the single LSTM-RNN.

In addition, we think some more details are worthy of being investigated further in the future: (1) It is meaningful to investigate the deep LSTM-RNN network, which should be trained with a large amount data. Well trained deep LSTM-RNN has been demonstrated to be more feasible in some applications. A deep LSTM-RNN will be implemented and presented in future; (2) many variants of RNN are published and have been demonstrated effectively in solving time series prediction problems. It is meaningful to further investigate and compare their performance, and find more preferable neural networks suitable for this particular application. Comparison of several popular variants of RNN will be presented in future.

## Figures and Tables

**Table 1:** Specifications of MSI3200 IMU (Inertial Measurement Unit).

**MEMS IMU**	**Gyroscope**	Range	±300°/s
		Bias stability (1σ)	≤10°/h
		Bias stability (Allan)	≤2°/h
		Angle random walk	≤10°/$\sqrt{h}$
	**Accelerometer**	range	±15 g
		Bias stability (1σ)	0.5 mg
		Bias stability (Allan)	0.5 mg
	**Power consumption**	1.5 W	
	**Weight**	250 g	
	**Size**	70 mm ×54 mm ×39 mm	
	**Sampling rate**	400 Hz	

**Table 2:** Standard deviation of Auto Regressive and Moving Average (ARMA) modeling results for the three-axis gyroscope.

	X	Y	Z
Raw data	0.0247	0.035	0.056
ARMA	0.017	0.028	0.042

**Table 3:** Standard deviation of the raw and predicted datasets by single LSTM-RNN.

	X	Y	Z
Raw data	0.0247	0.035	0.056
Single LSTM-RNN	0.0098	0.022	0.031

**Table 4:** Performance of gyroscope X-axis with varying values of input vector.

Length	STD	Time (s)
5	0.0096	1.25
10	0.0095	1.33
15	0.0063	2.13
20	0.0052	2.90
30	0.0094	2.98

**Table 5:** Specifications of LSTM-RNN.

Batch size	128
Training epoch	50
Learning rate	0.01
Hidden unit amount	1

**Table 6:** Comparison of Single LSTM-RNN and multi-layer LSTM-RNN.

	X			Y			Z		
	Training Loss	STD	Time	Training Loss	STD	Time	Training Loss	STD	Time
Single LSTM-RNN	0.00053	0.0098	4.52	0.0010	0.022	4.94	0.022	0.031	4.56
Multi-layer LSTM-RNN	0.000467	0.011	9.31	0.0009	0.023	9.21	0.014	0.038	8.65
Raw data	/	0.0246	/	/	0.0352	/	/	0.056	/

**Table 7:** Comparison of single LSTM-RNN with 50 training epochs and multi-layer LSTM-RNN with 20 training epochs.

	X			Y			Z		
	Training Loss	STD	Time	Training Loss	STD	Time	Training Loss	STD	Time
Single LSTM-RNN	0.00053	0.0098	4.52	0.0010	0.022	4.94	0.022	0.031	4.56
Multi-layer LSTM-RNN	0.00045	0.012	3.68	0.0009	0.024	3.82	0.017	0.038	3.76
Raw data	/	0.0246	/	/	0.0352	/	/	0.056	/

**Table 8:** Standard deviation of ARMA modeling results for the three-axis gyroscope.

	X	Y	Z
Raw data	0.0247	0.035	0.056
ARMA	0.017	0.028	0.042
Single LSTM-RNN	0.0098	0.022	0.031

**Table 9:** Maximum attitude errors from raw, ARMA and LSTM-RNN

	Pitch	Roll	Yaw
Raw data	−5.070	−1.952	−3.853
ARMA	−4.268	−1.601	−1.873
Single LSTM-RNN	−2.231	−0.942	0.826
